# Development and community-based validation of the IDEA study Instrumental Activities of Daily Living (IDEA-IADL) questionnaire

**DOI:** 10.3402/gha.v7.25988

**Published:** 2014-12-29

**Authors:** Cecilia Collingwood, Stella-Maria Paddick, Aloyce Kisoli, Catherine L. Dotchin, William K. Gray, Godfrey Mbowe, Sarah Mkenda, Sarah Urasa, Declare Mushi, Paul Chaote, Richard W. Walker

**Affiliations:** 1 The Medical School, Newcastle University, Newcastle upon Tyne, UK; 2 Northumbria Healthcare NHS Foundation Trust, North Tyneside General Hospital, North Shields, UK; 3 Institute of Neuroscience, Newcastle University, Newcastle upon Tyne, UK; 4 Hai District Medical Office, Hai, Tanzania; 5 Institute of Ageing, Newcastle University, Newcastle upon Tyne, UK; 6 Community Health Department, Kilimanjaro Christian Medical University College, Moshi, Tanzania; 7 Department of Medicine, Kilimanjaro Christian Medical Centre, Moshi, Tanzania; 8 Institute of Health and Society, Newcastle University, Newcastle upon Tyne, UK

**Keywords:** instrumental activities of daily living, validation, screening, dementia, Africa, Tanzania

## Abstract

**Background:**

The dementia diagnosis gap in sub-Saharan Africa (SSA) is large, partly due to difficulties in assessing function, an essential step in diagnosis.

**Objectives:**

As part of the Identification and Intervention for Dementia in Elderly Africans (IDEA) study, to develop, pilot, and validate an Instrumental Activities of Daily Living (IADL) questionnaire for use in a rural Tanzanian population to assist in the identification of people with dementia alongside cognitive screening.

**Design:**

The questionnaire was developed at a workshop for rural primary healthcare workers, based on culturally appropriate roles and usual activities of elderly people in this community. It was piloted in 52 individuals under follow-up from a dementia prevalence study. Validation subsequently took place during a community dementia-screening programme. Construct validation against gold standard clinical dementia diagnosis using DSM-IV criteria was carried out on a stratified sample of the cohort and validity assessed using area under the receiver operating characteristic (AUROC) curve analysis.

**Results:**

An 11-item questionnaire (IDEA-IADL) was developed after pilot testing. During formal validation on 130 community-dwelling elderly people who presented for screening, the AUROC curve was 0.896 for DSM-IV dementia when used in isolation and 0.937 when used in conjunction with the IDEA cognitive screen, previously validated in Tanzania. The internal consistency was 0.959. Performance on the IDEA-IADL was not biased with regard to age, gender or education level.

**Conclusions:**

The IDEA-IADL questionnaire appears to be a useful aid to dementia screening in this setting. Further validation in other healthcare settings in SSA is required.

The population of sub-Saharan Africa (SSA) is ageing rapidly with an associated increase in non-communicable diseases, such as dementia, presenting a challenge to already scarce healthcare and human resources. In 2013, there were estimated to be 1.31 million people with dementia in SSA, which will rise to a projected 5.05 million people by 2050 (1). Despite this, the diagnosis of dementia in many parts of SSA can be problematic due to a severe shortage of specialist physicians, such as neurologists, psychiatrists, and geriatricians (2, 3), and an estimated 200 times fewer trained mental health workers in SSA in comparison to European countries (2–4). The World Health Organization (WHO) have developed the Mental Health Gap Action Programme (mhGAP) (5) to help address some of the issues around identification and management of people with mental health problems in low- and middle-income countries (LMICs). In line with the mhGAP, the WHO recommended strategy for diagnosis and management of chronic disease and mental disorders in low-resource settings is one of task shifting. Task shifting aims to support and enable non-specialist and primary care workers to provide services delivered by specialists and physicians in higher resourced settings (6, 7). This approach requires use of clearly defined protocols alongside brief assessment tools designed and validated for use in these low-resource environments with high sensitivity and specificity to assist clinical decision making. Unfortunately, assessment tools for dementia designed for use in SSA are currently few, especially those designed for use by non-specialists in primary care.

Cognitive screening tools designed for use in SSA are currently few, despite the difficulties of assessing cognition in this predominantly low-literacy setting. The Community Screening Instrument for Dementia (CSI-D) has been previously validated in Nigeria (8) and Kenya (9) and used in research studies, but is too lengthy for routine screening. A brief CSI-D has been developed by the 10/66 research group from data collected as part of a series of prevalence studies (10). However, it has not yet been externally validated, and the data used for its development were from India, China, and Latin America where background education levels are likely to be much higher than in many areas of SSA. A brief screening instrument [the IDEA (Intervention for Dementia in Elderly Africans) cognitive screen] has been developed and validated by members of our team, specifically for use in SSA. It is intended to minimise educational bias (11).

Used alone, cognitive screening is not adequate as a clinical decision aid, even if using tools specifically designed for SSA. Poor performance may be due to physical illness, sensory impairment or lack of confidence rather than cognitive impairment or dementia. A collateral history from an informant is also required, and functional assessment tools are necessary to assist staff in identifying those with likely dementia, as well as forming a core part of the formal diagnostic criteria for dementia. Functional assessment is generally agreed to include two main elements: activities of daily living (ADLs) and instrumental (or extended) activities of daily living (IADLs). ADLs are basic self-care activities such as bathing, feeding, and dressing independently. Assessment of these is often useful in identifying care needs and dependence. IADLs are more complex activities generally agreed to be affected earlier in cognitive impairment as they require more intact neurocognitive abilities to complete (12).

A number of IADL assessment tools exist, with the most widely used being the Lawton IADL scale (12). Used alone, the Lawton IADL scale is reported to have a sensitivity of 0.85–0.90 and specificity of 0.66–0.98 in identifying dementia (13). Most IADL scales have been developed in high-income countries, and assume independent living. In LMICs, cultural norms and social roles differ, and existing IADL scales are often inappropriate, particularly where multigenerational living is common, and older people may not be directly responsible for household tasks measured on existing scales. Performance on other tasks may be restricted by lack of availability or access to amenities such as transport, or adherence to traditional gender roles. These issues in functional assessment are well recognised and have been previously thought to be responsible for falsely low dementia prevalence rates reported in LMICs, particularly those in SSA. Culturally appropriate IADL scales have been developed for use in LMICs (see Table 1). A culturally specific, semi-structured assessment of IADL to assist in dementia diagnosis has been developed in Nigeria, but must be conducted by a trained clinical assessor and requires a home visit (14).

**Table 1:** Instrumental activities of daily living scales devised for use in low- and middle-income countries

Author, year	Location, setting	Development method	Validation sample	Validation method	Key findings
Senanarong et al., 2003 (THAI-ADL) (28)	Thailand, community and specialist clinic	Specialist panel discussion	181 memory clinic attendees; mean age 69 years	Agreement with Thai MMSE FAQ, Barthel, CDR	Correlation with Thai MMSE (*r=*0.69), CDR (*r=*0.81), Barthel Index (*r=*0.80) and FAQ (*r=*0.88)
Jitapunkul et al., 1994 (Chula ADL) (29)	Thailand, community sample	Factor analysis of items from the Barthel index and Office of Populations Censuses and Surveys (OPCS) disability score	703 people aged 60 years and over; mean age 68 years	Agreement with OPCS and Barthel Index	Aim of scale was to measure disability appropriately in local population
Umayal et al., 2010 (30)	Sri Lanka, nursing home population	Validation of a modified Blessed dementia scale and Bristol ADL. Scores were modified by expert/clinician opinion	Nursing home residents aged 65 years and over; mean age 73 years	Dementia ICD-10 criteria by consultant psychiatrist	Modified Bristol scale: AUROC 0.933. Sensitivity 100%, specificity 74.2% Modified Blessed scale: AUROC 0.892. Sensitivity 100%, specificity 71%
Fillenbaum et al., 1999 (EASI) (31)	Kerala, India low-literacy community	Community discussion with elders and health workers related to usual social roles and activities of the elderly	Pilot testing 100 people, initial validation 387 people aged 55 years and over; mean age 69.5 years	Hindi MMSE score< 22	Cronbach's alpha = 0.82. Lower scores in females, older people, illiterate people and those with lower cognitive function
Mathuranath et al., 2005 (E-ADL) (32)	India memory clinic	Development and validation of scale based on Lawton IADL. Input from senior citizens group and clinicians on suitable IADL	Validation on 240 memory clinic attendees and 135 controls from background population	DSM dementia	AUROC 0.97. Sensitivity 0.91, specificity 0.99
Hendrie et al., 2006 (CHIF) (14)	Nigeria	Expert opinion of clinicians. Also took into account ‘items usually included in assessments of ADL’	Community sample of 295	DSM dementia Blessed dementia scale, MMSE	AUROC 0.925 for dementia Cronbach's alpha 0.83 Correlated with Blessed DS 0.56 and MMSE 0.44

## Aims

Our aim was to develop and validate a brief, and culturally appropriate, assessment of IADL, suitable for use by primary healthcare workers in identifying dementia in SSA when combined with a cognitive screening tool (15). It is hoped that, after completing cognitive and functional assessment, a village health worker would feel confident in choosing an appropriate referral pathway and be able to offer advice to patients and their families.

## Method

### Ethics

Ethical approval for the pilot fieldwork testing and subsequent validation was given by the National Institute of Medical Research, Dar-es-Salaam, Tanzania. Additional ethical approval for the validation work was given by Kilimanjaro Christian Medical University College, Moshi, Tanzania.

### Setting

The study was conducted in six villages in the Hai district of Tanzania. Tanzania is a low-income, developing country; the average life expectancy is 61 years, with around 5% of the population aged 65 or over (16). Hai district is largely rural, and situated at the base of Mount Kilimanjaro, in the north of the country. There are two government hospitals in Hai and numerous dispensaries and smaller health centres.

Since 1992, Hai has contained a demographic surveillance site (DSS) (17). The DSS had a population of 161,119 in 2009, most of whom are subsistence farmers (15). Each village within the Hai DSS has one or two healthcare workers or enumerators with responsibilities for carrying out regular population censuses and completing public health activities under supervision from the District Medical Officer. Enumerators reside within the villages for which they are responsible and are well-respected members of their community. They have considerable experience of research projects focussing on chronic diseases and older adults.

### Development of the IDEA-IADLs questionnaire

To facilitate the development of an IADL questionnaire for use in the IDEA study (the IDEA-IADL questionnaire), a workshop was held with all district enumerators and local healthcare workers. In total, 55 people attended the workshop. Prior to attending this workshop, the enumerators had received extensive training on dementia as part of a prevalence study conducted in 2010 (15). Additionally, enumerators had obtained practical experience of screening for dementia using the CSI-D. The workshop was facilitated by A.K. and S.-M.P.

It was explained to the group that assessment of functional impairment was reported to be difficult in traditional societies because assessment tools were generally designed for use in high-income countries with different cultural expectations of older individuals. The group was therefore asked to list, based on their experience in their local communities, those activities that would be expected of an elderly person, regardless of gender. It was stressed that this should be regardless of physical disability or sensory impairment (e.g. poor eyesight or hearing). To avoid biasing the responses obtained, no existing IADLs scales were discussed at the workshops.

Activities were suggested and then discussed with the wider group until a consensus was reached. The discussion was facilitated by a registered nurse with experience of working in the Hai district with people with dementia. It was felt that, in most circumstances, the family or community would attempt to assist the elderly person in carrying out roles or tasks that they found difficult, and therefore a graded response to each question, rather than a dichotomous (yes/no), answer would be more appropriate. No attempt was made to model answers upon an existing assessment scale or to modify an existing scale.

### Pilot fieldwork testing of the IDEA-IADL questionnaire

The IDEA-IADL questionnaire developed was piloted on 52 people identified and followed up as part of a dementia prevalence study (15). The scale was administered by a healthcare worker (nurse, clinical officer or assistant medical officer) who recorded responses from an informant (a close relative or friend of the participant). All healthcare workers who administered the questionnaire had been involved in the original development of the scale and subsequent discussion.

#### Diagnosis of dementia and mild cognitive impairment during pilot fieldwork testing

Once the IADL questionnaire had been administered, formal assessment for cognitive impairment was carried out by a research doctor (S-M.P.) who was blinded to the results of the IDEA-IADL questionnaire. All participants had a cognitive examination which included orientation, delayed recall, an assessment of attention and concentration (days of the week backwards), an assessment of language ability based on a structured conversation and ability to follow complex commands. A brief neurological and physical examination was also conducted and patients assessed regarding known dementia risk factors. Screening for depression was carried out using the geriatric depression scale (GDS) with further clinical assessment if indicated. An informant interview was completed in line with DSM-IV guidelines (18). Dementia diagnosis was based on DSM-IV dementia criteria (18). Mild cognitive impairment diagnosis was based on international consensus criteria (19).

### Community validation of the IDEA-IADL questionnaire

Participation in the study was on a voluntary, self-referral basis. A few days before the research team came to each village, information about the study, including what participation involved, was announced in local religious meetings and by local enumerators, with support from the village committee. Any residents aged 65 years or over wishing to take part, or any family members wishing to refer a relative, were invited to attend one of three screening days in each village. All participants were asked to attend with a relative or carer able to give an informant history. Eleven people were identified by village enumerators as too frail to attend screen and were visited at home.

Screening days took place at local village offices. Given the voluntary nature of participation, after dissemination of information about the study, consent was assumed for those who volunteered for screening. To avoid selection bias, any older people who wished to take part in the study, but were physically unable to attend screening days, were visited at home. Information was collected, on the participants’ gender, age, literacy, and educational level. Contact information for each participant was recorded to allow follow-up.

#### Functional assessments and cognitive screening

Function was assessed using the IDEA-IADL questionnaire and the Lawton IADL scale (12). Assessments were conducted by local healthcare workers who were blinded to all cognitive assessments. Both assessments were translated into Swahili and back translated prior to use.

Cognitive screening was conducted using the validated IDEA cognitive screen (11). The screen was administered by a local healthcare worker who was blind to the DSM-IV diagnosis and all functional assessments and who had attended a 4-day training course regarding dementia and use of the screening instruments. Subjects can score 0–15 points, with zero reflecting the lowest cognitive performance and 15 the highest cognitive performance. A score of 8–9 was taken to indicate possible dementia and a score of ≤7 probable dementia (11).

The assessors were asked to indicate on the study pro forma whether, in their opinion, the informant was felt to be reliable. The informant was considered unreliable if they were a non-relative (e.g. neighbour) who on questioning rarely came into contact with the participant and/or the information given by the informant largely contradicted the impression the interviewer had of the participant based on cognitive screening.

#### Diagnosis of dementia during validation

As during pilot fieldwork testing, DSM-IV diagnosis was provided by a UK-based research psychiatrist (S-M.P.) who was blind to the results of the cognitive and functional assessment conducted by the healthcare workers. Where diagnoses were in doubt, cases were discussed with a UK-based consultant in old age psychiatry.

Based on their IDEA cognitive screen scores, a stratified sample was followed up and underwent full clinical diagnostic assessment. The aim was to assess all those with probable and possible dementia as well as a randomly selected 10% of those with no dementia (scoring > 9). Random selection involved blindly picking participant's numbers out of a container.

Informants who failed to attend for a full diagnostic assessment interview were asked to attend on a subsequent day and, where this was not possible, were followed up on home visits by the clinician.

### Statistical methods

#### Data analysis

Statistical analysis was conducted using Microsoft Excel 2010 and IBM SPSS statistics version 2.1. All data were found to be non-normally distributed and therefore summarised in terms of median, inter-quartile range (IQR), and range. The significance level was set at 5% and two-tailed significance tests used throughout. Cronbach's alpha was used as a measure of internal consistency and factor analysis used to investigate underlying latent traits within the scale. For factor analysis, the method of principal components was used and a varimax rotation applied to aid data analysis. Area under the receiver operating characteristic (AUROC) curve was calculated to give an overall assessment of the predictive ability of each of the scales, with presence of clinical dementia (yes or no) as the state variable.

To identify the most appropriate weighting to give to the IDEA cognitive screen and IDEA-IADL questionnaire when combined into a single measure, binary logistical regression analysis was performed using the screening instrument scores as covariates and presence of clinical dementia (yes or no) as the outcome variable. Regression coefficients were converted to weights using the method of Sullivan et al. as part of their work on the Framingham Study (20). Linear regression was used to investigate the influence of age, gender, and education level on IDEA-IADL scores after adjusting for the influence of dementia diagnosis. The model validity was assessed by examination of eigenvalues, studentised residuals, and tolerance.

There were very few missing values and these appeared to be missing completely at random and non-informative. No attempt was made to impute these data, and these data were omitted from the analysis.

## Results

**Fig. 1:**
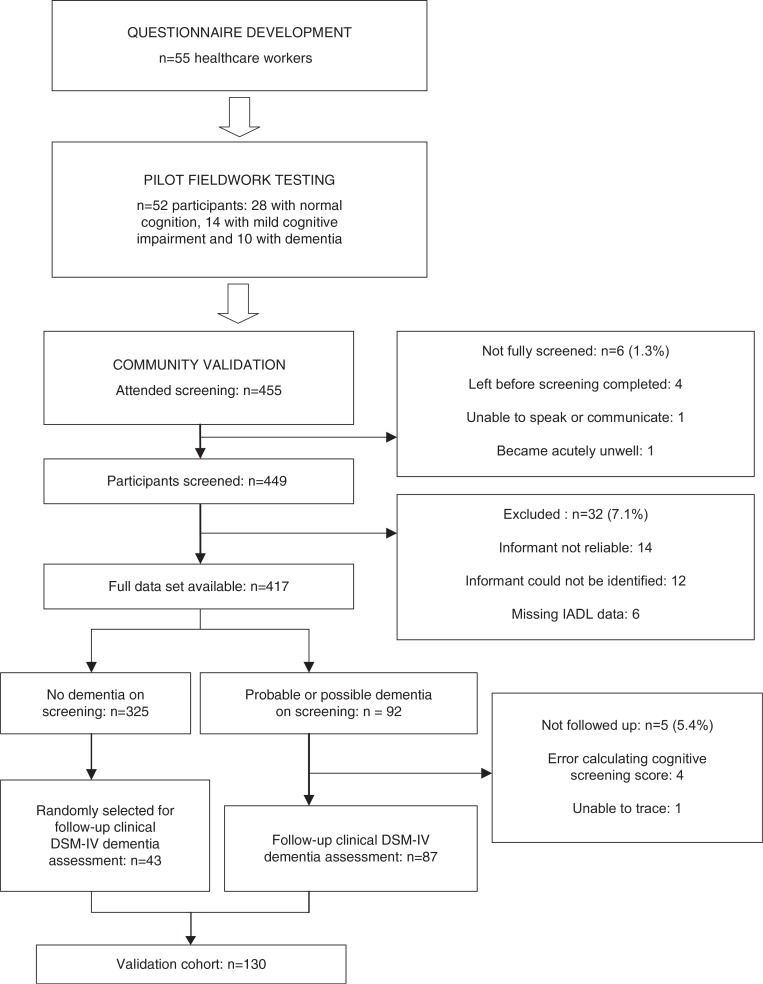
Flow diagram of the IDEA-IADL development and validation process.

A flow diagram summarising the steps in the development, and validation of the IDEA-IADL questionnaire is shown in Fig. 1.

### Development of the IDEA-IADL questionnaire

Health workers and enumerators from 52 villages within the DSS took part in the workshop. In addition, a number of other health workers involved in research projects attended the workshop including the community psychiatric nurse for the Hai district, and assistant medical officers and clinical officers with public health responsibilities.

A questionnaire consisting of 12 questions each scored from zero (‘cannot do this’) to three (‘can do it with no problems, do not need help’) was constructed based on the responses from participants. The questions included are shown in Table 2. The questionnaire was developed in Swahili and later translated into English for those team members not familiar with Swahili.

**Table 2:** Twelve questions initially included in the scale

	Swahili	English translation
1	Wanatoa historia	They give histories of the family, their life, past events
2	Wana suluhisha	They settle conflicts
3	Wanasaidia shughuli ndogo ndogo	They assist in small works in the home
4	Wanatoa ushauri	They give advice
5	Wanadumisha na kufundisha mila/unyago	They teach the traditions of society
6	Ni walinzi wa nyumbani	They watch over the house when others are out
7	Wanatunza wajukuu	They look after the grandchildren
8	Wanatoa ushawishi	Persuasion or changing people's ideas for the better
9	Wanasaidia katika maswala mazito kama sherehe	They preside over feasts and ceremonies
10	Wanapangia watu majukumu	Delegation of responsibilities to others
11	Wanasimamia haki	They fight for justice within the family and the community. They ensure fairness
12^a^	Wanafanya mirathi	They make their testament and decide on division of possessions after they have gone

a After pilot fieldwork, this question was removed for the scale because many people were unwilling to answer questions on this sensitive topic.

### Pilot fieldwork testing

Of the 52 participants in pilot fieldwork testing of the IDEA-IADL questionnaire, 28 had normal cognition, 14 had mild cognitive impairment, and 10 had dementia. It rapidly became evident that one item initially included on the scale (‘they make their will and testament and make decisions on their property after they have gone’) was not suitable for inclusion in the scale as some health workers were uncomfortable asking this question, although they felt it was an important part of an elderly person's role. The question was only answered by 22 participants (42.3%) and was not included in subsequent analyses. No attempt was made to replace the item with another question as it was felt all items of interest had been considered.

Cronbach's alpha for the remaining 11 questions was 0.904, indicating high internal consistency. When the presence of dementia was used as the outcome variable, the AUROC curve was 0.814 [95% confidence interval (CI) 0.689–0.939] for the questionnaire. At a cut-off of ≤23 as indicative of dementia, sensitivity was 80.0% and specificity 78.6%.

### Validation

Of a total 455 who presented for screening, six were excluded (one unable to speak or communicate, one due to acute medical illness requiring immediate hospital admission, and four left the screening event before screening was completed). For the latter four participants, it was assumed that consent had been withdrawn and they were excluded from all analyses. Of the 449 participants for who data were collected, 32 (7.1%) were excluded from the study after screening data was collected. Fourteen (3.1%) were excluded because the informant was deemed unreliable in the opinion of the interviewers, 12 (2.7%) were excluded because a reliable informant could not be identified, and six (1.3%) were excluded due to incomplete IADL data. Thus, complete IDEA-IADL questionnaire and Lawton IADL scale scores were available for 417 subjects (see Fig. 1).

For the IDEA-IADL questionnaire, Cronbach's alpha was 0.959 indicating high internal consistency. Factor analysis revealed only one factor with an eigenvalue greater than one, explaining 71.6% of the variance in the model. Table 3 shows the component matrix for the first three factors, their eigenvalues, and the percentage of variance explained by each component. The questionnaire took a median of 5 min (IQR 3–7, data available for 320 people) to administer.

**Table 3:** Factor analysis of IDEA-IADL scores

	Component		
	
	1	2	3
Eigenvalue	7.881	0.718	0.411
Variance explained (%)	71.6	6.5	3.7
Factor loadings			
Question 1 (give histories)	0.854	−0.245	0.027
Question 2 (settle conflicts)	0.864	−0.165	−0.016
Question 3 (assist in house)	0.784	0.411	0.300
Question 4 (give advice)	0.895	−0.135	−0.119
Question 5 (teach traditions)	0.865	−0.227	0.127
Question 6 (watch over house)	0.782	0.399	−0.322
Question 7 (childcare)	0.781	0.441	0.026
Question 8 (persuade others)	0.876	−0.123	−0.0182
Question 9 (preside over ceremonies)	0.832	−0.080	0.365
Question 10 (delegate)	0.892	−0.026	−0.057
Question 11 (ensure fairness)	0.876	−0.127	−0.126

Scores for the IDEA cognitive screen are summarised in Table 4, together with demographic data. Ninety-two of the 97 people who scored ≤9 (possible or probable dementia) had IADL data available (see Table 4 and Fig. 1). Of these, one could not be traced for a follow-up and four were not followed up due to addition errors in their scores, such that they were initially thought to have scored >9. Thus, 87 people with possible or probable dementia were followed up and clinically assessed for dementia by the study doctor. A further 43 randomly selected people who scored >9 were also followed up and clinically assessed. This gave a validation cohort of 130 (see Fig. 1), 35 (26.9%) of whom were diagnosed with DSM-IV dementia. With the presence of dementia used as the outcome measure, AUROC curves for the IDEA cognitive screen, the IDEA-IADL questionnaire, and the Lawton IADL scale are summarised in Table 5.

**Table 4:** IDEA cognitive screen scores

	Score≤7	Score 8 or 9	Score≥10
Number	40	57	352
Median age (IQR)	80 (73.75–85.5)	76 (70–81.25)	72 (67–79)
Number of females (%)	34 (85.0%)	41 (71.9%)	178 (50.6%)
Number with some formal education (%)	18 (47.4%), 2 missing values	34 (60.7%), 1 missing value	305 (87.6%), 4 missing values
Median Lawton scale score	3 (1–7), 3 missing values	6 (4–7), 2 missing values	7 (5.5–8), 27 missing values
Median IDEA-IADL questionnaire score	13 (4–30), 3 missing values	27 (15–32), 2 missing values	33 (27.5–33), 27 missing values
Seen for DSM-IV diagnostic assessment	36 (90.0%)	51 (89.5%)	43 (12.2%)
Diagnosis of dementia	21 (52.5%)	13/51 (25.5%)	1/43 (2.3%)

**Table 5:** AUROC curve analysis for the IDEA-IADL questionnaire, the Lawton IADL scale and the IDEA cognitive screen

	AUROC curve (95% CI)
Lawton IADL scale	0.828 (0.751–0.906)
IDEA-IADL questionnaire	0.896 (0.842–0.951)
IDEA cognitive screen	0.846 (0.776–0.915)
Combined IDEA cognitive screen and IDEA-IADL questionnaire	0.937 (0.896–0.979)

Logistic regression analysis indicated that, if combined as a singe measure, the IDEA cognitive screen and the IDEA-IADL questionnaire items should be weighted in a ratio of 5:1, respectively, as shown in Table 6. After doing this, the AUROC curve for the combined score was calculated and is shown in Table 5. Although both screening tools performed well independently, combined use of the IDEA cognitive screen and IDEA-IADL questionnaire resulted in an AUROC curve of 0.937 (0.896–0.979), compared with cognitive screening only (0.846; 95% CI 0.776–0.915) or IDEA-IADL alone (0.896; 95% CI, 0.842–0.951).

**Table 6:** Regression analysis

	β Coefficient	Scaled β coefficient	Exp β	Significance
Logistic regression model				
Total IDEA-IADL questionnaire	−0.0138	1	0.871 (95% CI 0.822 to 0.922)	<0.001
Total IDEA cognitive screen	−0.646	4.681	0.524 (95% CI 0.371 to 0.739)	<0.001
Constant	6.915	–	–	–
Linear regression model				
Any formal education	1.466 (95% CI −1.905 to 4.838)	–	–	0.391
Male gender	1.215 (95% CI −2.239 to 4.669)	–	–	0.487
Age (years)	−0.031 (95% CI −0.204 to 0.142)	–	–	0.725
DSM-IV dementia present	−15.896 (95% CI −19.978 to −11.815)	–	–	<0.001
Constant	26.900	–	–	–

Finally, linear regression was used to investigate whether the IDEA-IADL questionnaire was biased to age, gender, or education. Dementia diagnosis (yes or no) was included in the model since it is known to be strongly associated with each of these variables. As shown in Table 6, only dementia diagnosis emerged as an independent predictor of IDEA-IADL score, suggesting the questionnaire to be unbiased to each of the other variables.

## Discussion

Efficient and effective identification of people with dementia is an important first step in reducing the diagnosis and treatment gap that exists in many countries in SSA. A case-finding approach, in conjunction with a basic training programme on recognition of dementia, has been employed in similar low-resource settings, including Brazil (21) and India (22). This approach has had only moderate success, in part, due to the low overall prevalence of dementia in the community. Our suggested approach, based on validated screening tools, is likely to be more effective and would be in keeping with the protocol driven WHO mhGAP strategy (5).

The aim of this study was to develop an IADL questionnaire that could act as a clinical decision aid in a low-resource setting. Impairment in IADLs has been found to be predictive of later dementia in large population studies in the USA and France over periods of up to 10 years, irrespective of cognitive assessment scores within the normal range at baseline. In fact, IADL assessment scales have been used alone in population screening for dementia (13, 23). It has been suggested that these are less educationally biased than cognitive assessments in low-literacy settings (24, 25).

Previous IADL assessment instruments tend to be of three types: self-report by patient, informant interview, and direct observation (26, 27). All have their flaws, with the usefulness of self-report and informant interview limited by the cognitive ability of the patient and the reliability of the informant, respectively. Direct observation is often seen as a gold standard, although it can be resource-intensive and require a great deal of staff training, making it generally unsuitable for use in our setting. We have pragmatically chosen to develop a questionnaire that relies on informant interview. In a community setting, finding a reliable informant is usually possible and in the current study, only 3.1% (14 of 449) of informant interviews were considered unreliable by the interviewer.

Although a small number of functional assessment scales have been developed and validated for use in other LMICs (see Table 1), of these, only a proportion addressed face validity issues by involving the local population in development (31, 32). The remainder used clinician opinion for development or modification of scales designed in high-income countries.

The questionnaire worked well and had good predictive properties, both when used in isolation or together with a brief cognitive screening instrument. In a low-education, developing world, setting the increased predictive ability of cognitive screening, if combined with a functional assessment, has been noted by other authors (33). Involvement of local healthcare workers in the development of the questionnaire ensured good face validity. Furthermore, by focussing on normal social roles and activities, we hoped to minimise gender and education bias in the items included.

Internal consistency of the final questionnaire was high, indicating that all 11 items are broadly testing the same underlying trait. It could be argued that, because of such strong correlation between items, some may be redundant in the context of a single score-generating screening tool. However, the use of this tool in clinical practice needs to be considered. With increased number of IADLs assessed and explored with the informant, the tool will be more useful to healthcare staff in their overall clinical judgement than a tool that was designed to generate the most efficient statistical score. Considering this, alongside the fact that the time taken to complete the assessment is short and viable for screening use in every day clinical practice, it would seem more appropriate to include all 11 items.

We did not attempt to adjust responses to allow for the fact that some participants may not routinely engage in some of the activities included in the scale. Our main aim was to develop a screening instrument for use by non-specialist healthcare workers that would allow referral to specialist services as appropriate. We felt that a relatively straightforward scoring system would make the collection and interpretation of information relatively efficient. Further details on normal functioning could be obtained during a more detailed clinical assessment in those thought suitable for further assessment. The responsiveness of the IDEA-IADL questionnaire to changes in function will be assessed during future fieldwork.

### Limitations

A potential significant selection bias was possible as participation in the study involved self or family referral to screening at a village office. Those who were physically unwell or disabled were thus much less likely to attend for screening, and this could have significantly affected results since they are also more likely to perform poorly on functional assessment. To partly overcome this problem, we offered home visits for screening to patients identified as frail by village enumerators. Eleven participants were recruited in this way. Nevertheless, in this resource-limited setting, where primary healthcare coverage is very limited, such screening events are likely to be one of the most effective and sustainable methods of identifying people with cognitive impairment. As such, those included in this study are likely to be representative of one of the main groups the scale is designed to assess in normal use.

Five people achieving an IDEA cognitive screen score ≤9 were not seen for full clinical diagnosis. As this was a relatively small proportion of those seen, and they were missed randomly due to errors in addition on the part of research staff or contact and tracing difficulties, this is unlikely to have resulted in a substantial bias. For a further five participants, it was not possible to trace a reliable informant or informant data was incomplete. This has implications in clinical practice in that, for a small minority of people, it may not be possible to carry out a reliable functional assessment, hindering diagnosis.

Results can only be said to be representative of the community-dwelling population of Hai district. Further validation of the questionnaire in different geographical (e.g. urban and rural) and clinical (e.g. inpatients and outpatients) settings is required. As such, we are reluctant to extrapolate our findings beyond the setting it was developed and tested in. However, the IDEA-IADL questionnaire may be of use in assessing IADLs in other patient settings, in other parts of Tanzania and SSA and in other world regions.

Finally, cognitive and IADL assessments were carried out by nine separate assessors. Inter-rater reliability has yet to be established, and we cannot rule out the possibility that this may have influenced our results. Nevertheless, we have attempted to validate our tool in the setting for which it was designed, utilised by non-specialist primary healthcare workers in the community. We plan to assess the inter-rater reliability of the scale as an important next step.

## Conclusions

This is the first validation of functional assessment tools for use in Tanzania and one of very few conducted in SSA. The IDEA-IADL has good internal consistency and construct validity against the gold standard DSM-IV diagnosis of dementia. It is both time and cost-efficient, it does not require a specialist healthcare background to administer, and little training is needed. It appears to represent a better functional assessment in this population than the Lawton assessment.

## References

[1] Prince M, Guerchet M, Prina M (2013). Policy brief for heads of government: the global impact of dementia 2013–2050.

[2] Dotchin CL, Akinyemi RO, Gray WK, Walker RW (2013). Geriatric medicine: services and training in Africa. Age Ageing.

[3] Bower JH, Zenebe G (2005). Neurologic services in the nations of Africa. Neurology.

[4] Saxena S, Thornicroft G, Knapp M, Whiteford H (2007). Resources for mental health: scarcity, inequity, and inefficiency. Lancet.

[5] World Health Organization (2010). mhGAP Intervention Guide for mental, neurological and substance use disorders in non-specialized health settings.

[6] Beaglehole R, Epping-Jordan J, Patel V, Chopra M, Ebrahim S, Kidd M (2008). Improving the prevention and management of chronic disease in low-income and middle-income countries: a priority for primary health care. Lancet.

[7] Eaton J, McCay L, Semrau M, Chatterjee S, Baingana F, Araya R (2011). Scale up of services for mental health in low-income and middle-income countries. Lancet.

[8] Hall KS, Gao SJ, Emsley CL, Ogunniyi AO, Morgan O, Hendrie HC (2000). Community screening interview for dementia (CSI ‘D’); performance in five disparate study sites. Int J Geriatr Psychiatry.

[9] Chen CH, Mizuno T, Elston R, Kariuki MM, Hall K, Unverzagt F (2010). A comparative study to screen dementia and APOE genotypes in an ageing East African population. Neurobiol Aging.

[10] Prince M, Acosta D, Ferri CP, Guerra M, Huang Y, Jacob KS (2011). A brief dementia screener suitable for use by non-specialists in resource poor settings – the cross-cultural derivation and validation of the brief Community Screening Instrument for Dementia. Int J Geriatr Psychiatry.

[11] Gray WK, Paddick SM, Kisoli A, Dotchin CL, Longdon AR, Chaote P (2014). Development and validation of the identification and Intervention for Dementia in Elderly Africans (IDEA) study dementia screening instrument. J Geriatr Psychiatry Neurol.

[12] Lawton MP, Brody EM (1969). Assessment of older people: self-maintaining and instrumental activities of daily living. Gerontologist.

[13] Castilla-Rilo J, Lopez-Arrieta J, Bermejo-Pareja F, Ruiz M, Sanchez-Sanchez F, Trincado R (2007). Instrumental activities of daily living in the screening of dementia in population studies: a systematic review and meta-analysis. Int J Geriatr Psychiatry.

[14] Hendrie HC, Lane KA, Ogunniyi A, Baiyewu O, Gureje O, Evans R (2006). The development of a semi-structured home interview (CHIF) to directly assess function in cognitively impaired elderly people in two cultures. Int Psychogeriatr.

[15] Longdon AR, Paddick SM, Kisoli A, Dotchin C, Gray WK, Dewhurst F (2013). The prevalence of dementia in rural Tanzania: a cross-sectional community-based study. Int J Geriatr Psychiatry.

[16] World Bank (2014). Data: Tanzania. http://data.worldbank.org/country/tanzania#cp_wdi.

[17] Adult Morbidity and Mortality Project (AMMP) (2004). Policy implications of adult morbidity and mortality.

[18] American Psychiatric Association (1994). Diagnostic and statistical manual of mental disorders.

[19] Winblad B, Palmer K, Kivipelto M, Jelic V, Fratiglioni L, Wahlund LO (2004). Mild cognitive impairment – beyond controversies, towards a consensus: report of the International Working Group on Mild Cognitive Impairment. J Intern Med.

[20] Sullivan LM, Massaro JM, D'Agostino RB (2004). Presentation of multivariate data for clinical use: the Framingham study risk score functions. Stat Med.

[21] Ramos-Cerqueira AT, Torres AR, Crepaldi AL, Oliveira NI, Scazufca M, Menezes PR (2005). Identification of dementia cases in the community: a Brazilian experience. J Am Geriatr Soc.

[22] Shaji KS, Arun Kishore NR, Lal KP, Prince M (2002). Revealing a hidden problem. An evaluation of a community dementia case-finding program from the Indian 10/66 dementia research network. Int J Geriatr Psychiatry.

[23] Juva K, Makela M, Erkinjuntti T, Sulkava R, Ylikoski R, Valvanne J (1997). Functional assessment scales in detecting dementia. Age Ageing.

[24] Barberger-Gateau P, Commenges D, Gagnon M, Letenneur L, Sauvel C, Dartigues JF (1992). Instrumental activities of daily living as a screening tool for cognitive impairment and dementia in elderly community dwellers. J Am Geriatr Soc.

[25] Iavarone A, Milan G, Vargas G, Lamenza F, De Falco C, Gallotta G (2007). Role of functional performance in diagnosis of dementia in elderly people with low educational level living in Southern Italy. Aging Clin Exp Res.

[26] Gold DA (2012). An examination of instrumental activities of daily living assessment in older adults and mild cognitive impairment. J Clin Exp Neuropsychol.

[27] Sikkes SA, de Lange-de Klerk ES, Pijnenburg YA, Scheltens P, Uitdehaag BM (2009). A systematic review of Instrumental Activities of Daily Living scales in dementia: room for improvement. J Neurol Neurosurg Psychiatry.

[28] Senanarong V, Harnphadungkit K, Prayoonwiwat N, Poungvarin N, Sivasariyanonds N, Printarakul T (2003). A new measurement of activities of daily living for Thai elderly with dementia. Int Psychogeriatr.

[29] Jitapunkul S, Kamolratanakul P, Ebrahim S (1994). The meaning of activities of daily living in a Thai elderly population: development of a new index. Age Ageing.

[30] Umayal S, Kulathunga M, Somaratne S, Srikanth S, Kathriarachchi S, De Silva R (2010). Validation of a functional screening instrument for dementia in an elderly Sri Lankan population: comparison of modified Bristol and blessed activities of daily living scales. BMC Res Notes.

[31] Fillenbaum GG, Chandra V, Ganguli M, Pandav R, Gilby JE, Seaberg EC (1999). Development of an activities of daily living scale to screen for dementia in an illiterate rural older population in India. Age Ageing.

[32] Mathuranath PS, George A, Cherian PJ, Mathew R, Sarma PS (2005). Instrumental activities of daily living scale for dementia screening in elderly people. Int Psychogeriatr.

[33] Jacinto AF, Brucki SM, Porto CS, Martins Mde A, Citero Vde A, Nitrini R (2014). Suggested instruments for General Practitioners in countries with low schooling to screen for cognitive impairment in the elderly. Int Psychogeriatr.

